# Factors associated with long-term mechanical ventilation in patients undergoing cardiovascular surgery

**DOI:** 10.1186/s12872-023-03315-7

**Published:** 2023-05-25

**Authors:** Shahram Rahimi, Alireza Abdi, Nader Salari, Shamarina Shohaimi, Mehran Naghibeiranvand

**Affiliations:** 1 Student Research Committee, Kermanshah University of Medical Sciences, Kermanshah, Iran; 2 Department of Emergency and Critical Care Nursing, Faculty of Nursing and Midwifery, Kermanshah University of Medical Sciences, Kermanshah, Iran; 3 Department of Biostatistics, Faculty of Nursing and Midwifery, Kermanshah University of Medical Sciences, Kermanshah, Iran; 4 Department of Biology, Faculty of Science, University Putra Malaysia, Serdang, Selangor Malaysia; 5 Department of Nursing, Khorramabad Branch, Islamic Azad University, Khorramabad, Iran

**Keywords:** Long-term mechanical ventilation, Cardiovascular surgery, Critical care, Nursing

## Abstract

**Background:**

One of the main therapy for coronary artery disease is surgery. Prolonged mechanical ventilation in patients with cardiac surgery is associated with high mortality. This study aimed to determine the factors related to long-term mechanical ventilation (LTMV) in patients undergoing cardiovascular surgery.

**Methods:**

The present study was a descriptive-analytical study in which the records of 1361 patients who underwent cardiovascular surgery and were on a mechanical ventilator during 2019–2020 at the Imam Ali Heart Center in Kermanshah city were examined. The data collection tool was a three-part researcher-made questionnaire including demographic characteristics, health records, and clinical variables. Data analysis was done using descriptive and inferential statistical tests and SPSS Version 25 software.

**Results:**

In this study, of the 1361 patients, 953 (70%) were male. The results indicated that 78.6% of patients had short-term mechanical ventilation, and 21.4% had long-term mechanical ventilation. There was a statistically significant relationship between the history of smoking, drug use, and baking bread with the type of mechanical ventilation (*P* < 0.05). Also, based on the regression test, some parameters, such as the history of respiratory conditions, could predict the prolongation of mechanical ventilation. Creatinine levels before surgery, chest secretions after surgery, central venous pressure after surgery, and the status of cardiac enzymes before surgery also affect this issue.

**Conclusion:**

This study investigated some factors related to prolonged mechanical ventilation in patients undergoing heart surgery. For optimizing the care and therapeutic measures, It is suggested, healthcare workers have a detailed assessment on patients with factors such as the history of baking bread, history of obstructive pulmonary disease, history of kidney disease, use of an intra-aortic pump, number of respirations and systolic blood pressure 24 h after surgery, creatinine level 24 h after surgery, chest secretions after surgery, and the amount of pre-operative ejection fraction and cardiac enzymes (CK-MB).

## Background

More than sixteen million people die each year from cardiovascular disease (CVD), which is currently the most prevalent severe, chronic, and life-threatening disease in the world. More than eighty percent of these deaths are in low and middle-income countries [1]. Out of every 700–800 daily deaths in Iran, 317 cases are due to cardiovascular diseases. This disease is regarded as the leading cause of disability in society, and massive economic costs are spent to identify, treat and care for those affected [2]. Coronary artery disease has been identified as the leading cause of death, and it can be caused by various risk factors, the most important of which is atherosclerosis. Surgery is one of the primary treatments for coronary artery disease [3].

In Iran, 30,000 heart surgeries are performed annually, of which 50 to 60 percent are for coronary artery bypass surgery (CABG). The average estimated cost of surgery for each patient is between 7 and 10 million tomans (approximately 400$) [4]. The saphenous veins or mammary arteries are used to create an alternative vessel path to one or more coronary arteries, as well as a shortcut path to supply blood to the heart muscles [5]. These patients are usually on mechanical ventilation (MV) for less than 6 or 8 h; however, it can take up to 24 h to wean these patients [6, 7].

In patients under MV, the device should be disconnected as soon as possible. On the other hand, patients with dependency on MV need specialized care, frequent monitoring, and long-term hospitalization in intensive care units, which increases the costs of patients and bed occupation of units. For these reasons, timely and safe weaning from MV leads to optimum results for patients [8]. Several factors influence patient weaning from MV, including cardiorespiratory status, nervous system, nutritional status, psychological status, and other physiological factors. In patients under MV, the stability of physiological indicators is one of the conditions for weaning [9]. The evaluation for weaning readiness is done both objectively and subjectively. The mental evaluation includes lack of agitation, awareness of time and place, no use of sedatives, an effective cough, no sweating, and no paradoxical breathing, and the objective evaluation includes gas exchange and hemodynamic stability. Gas exchanges include arterial blood oxygen saturation greater than or equal to 90%, arterial blood oxygen pressure greater than or equal to 60 mm Hg, pH of 7.35 to 7.45, and arterial carbon dioxide pressure less than 50, as well as hemodynamic stability (heart rate out of 100, systolic blood pressure less than 180 and greater than 90 mmHg, and respiratory rate less than or equal to 25) [10].

Many studies have found that rapid and early weaning from MV in most heart surgery patients is safe, and it is performed in almost all cardiothoracic units worldwide because of its benefits. In a study on the effects of early weaning on cardiopulmonary function, Gal et al. found that the increase in the filling of the left ventricle, the improvement of the function of the ventricles, and the increase in cardiac output are among the positive results of early weaning. On the other hand, this has beneficial effects on the respiratory system, such as lowering the risk of hospital-acquired pneumonia and lung tissue damage [11].

Based on the studies conducted by Ji et al. (2010) and Saleh et al. (2012), age impaired the duration of MV. While in the study of Gomes (2015) and Sislagh (2007), there is no relationship between age and duration of MV [7, 12, 13]. There was no consensus on gender; Ingersoll (1991) and Doering (2001) believe gender does not affect weaning time, whereas Ghanbari et al. (2018) claim that weaning time is longer in women than in men [14–16]. Hassanzadeh et al. (2016) showed no significant relationship between body mass index and the duration of mechanical ventilation [17].

Although numerous studies have been conducted on the causes of LTMV and contradictory results have been obtained, there is still no adequate summary of the impact of related factors on this issue after coronary artery bypass surgery, and in the majority of these studies, few variables have been mentioned. The present study aimed to determine the factors related to long-term mechanical ventilation (LTMV) in patients undergoing heart surgery.

## Methods

The present study was a descriptive-analytical study in which the records of all patients undergoing coronary artery bypass surgery and MV admitted to the cardiac intensive care unit of Imam Ali Heart Center (AS) in Kermanshah during the years 2019–2020 were examined. The case group was defined as the patients undergoing coronary artery bypass surgery who were on MV for more than 24 h, and the control group was the patients on MV for less than 24 h. According to several studies, long-term mechanical ventilation (LTMV) occurs when patients undergoing cardiovascular surgery are subjected to MV for more than 24 h [6, 7]. Out of 1572 patients who underwent cardiovascular surgery, the records of 1361 eligible patients were examined. The sampling method was census (complete enumeration). The inclusion criteria were patients over 18 years of age and under MV after cardiovascular surgery. The exclusion criteria included patients requiring re-surgery or having incomplete records.

After obtaining permission from the Ethics Committee of Kermanshah University of Medical Sciences, receiving an introduction letter from the Vice-chancellor of Research and Technology of the University, and presenting it to the hospital authorities, data collection was commenced. Since the study design was retrospective, the researcher collected information from the records archive unit of the hospital and referred to the records of all patients hospitalized in open heart intensive care units undergoing coronary bypass surgery for two years from 2019 to 2020. Therefore, they were subjected to deliberate review. The duration of MV was calculated from the moment the patient entered the open-heart intensive care unit from the operation room until the time of the endotracheal tube extubation. 

The required information was extracted from patients' records and entered into a researcher-made checklist. In cases with incomplete information, the researcher called the patient or family and attached additional details to the checklist.

The data collection tool was a researcher-made questionnaire (information collection form) organized into three parts. The first part of the questionnaire included demographic variables, including age, gender, marital status, education, place of residence, height, weight, and body mass index. The second part details the variables of health records, including the history of diabetes, high blood pressure, high blood lipids, heart disease, central nervous system diseases, chronic kidney diseases and lung diseases, glandular diseases, history of open-heart surgery, smoking, drug use and alcoholic beverages, and history of baking bread. The third part of the clinical variable included factors before and after surgery. Factors before surgery include systolic and diastolic blood pressure, heart rate, respiration rate, arterial blood oxygen saturation percentage, arterial blood carbon dioxide pressure, ejection fraction, creatinine, cardiac enzyme (CK-MB), and hemoglobin before surgery. Factors during surgery incorporate anesthetic drugs, duration of being on the pump, on/off pump surgical method, simultaneous valve repair, number and type of transplanted vessels, and post-surgery factors, including recording the average of the first 24 h of variables; respiratory rate, heart rate, systolic and diastolic blood pressure, chest secretions, central venous pressure, creatinine, urinary output, hemoglobin, arterial blood oxygen pressure, arterial blood carbon dioxide pressure, use of heart-stimulating and heart-decreasing drugs, dysrhythmias, the length of time the endotracheal tube. To determine the validity of the questionnaire, the content validity method was used in such a way that the instrument was given to ten members of the academic staff of the nursing department, two people from the anesthesiology department, two cardiologists, and eight nurses working in the cardiac intensive care unit. Herewith their recommendations were obtained and applied to the instrument.

Descriptive statistical methods of frequency, percentage, mean and standard deviation were used. For comparing demographic variables in two groups of short-term MV and LTMV, the Chi-square test and comparing clinical variables and history of underlying diseases according to the type of short-term MV and LTMV, an independent t-test was used. Linear regression was applied to guide the predictability of LTMV by significant variables. Data analysis was done using SPSS Version 25 software. The significant level of tests was 0.05.

## Results

In this study, 1361 patients undergoing cardiovascular surgery were examined, whose minimum age was 35 and maximum 87 years; 953 (70%) were male, 1298 (95.4%) were married, and 621 (45.6%) were illiterate, 1101 (80.9%) were city residents. Also, 293 (21.5%) had a history of drug use, 453 (33.3%) had a history of smoking, 28 (2.1%) had a history of alcohol consumption, and 503 (37%) were blood type A. The result of the Chi-square test showed that there was a statistically significant relationship between smoking history, drug use, and bread-baking history with the type of MV (*P* < 0.05) (Table 1).

**Table 1:** Relationship between demographic variables and MV status

Demographic variables	MV status	Short mechanical ventilation	Long mechanical ventilation	Chi-square test statistic	*P*-value
		Frequency (percentage)	Frequency (percentage)		
Sex	Man	753 (79.0)	200 (21.0)	0.295	0.587
	Female	317 (77.7)	91 (22.3)		
Marital status	Married	1024 (78.9)	274 (21.1)	1.23	0.267
	Single	1(25)	3 (75)		
	Divorced	12 (92.3)	1 (7.7)		
	Widow	33 (71.7)	13 (28.3)		
Education	Unknown	72 (72)	28 (28)	4.43	0.613
	Illiterate	485 (78.1)	136 (21.9)		
	Elementary	206 (81.1)	48 (18.9)		
	High school	130 (77.8)	37 (22.2)		
	Diploma	131 (81.4)	30 (18.6)		
	College Education	46 (79.3)	12 (20.7)		
Life location	City	868 (78.8)	233 (21.2)	0.169	0.919
	Village	184 (77.6)	53 (22.4)		
	Foreigners	18 (78.3)	5 (21.7)		
Smoking history	No	728 (80.2)	180 (19.8)	3.93	0.028*
	Yes	342 (75.5)	111 (24.5)		
Drug use history	No	852 (79.8)	216 (20.2)	3.94	0.047*
	Yes	218 (74.4)	75 (25.6)		
History of alcohol consumption	No	1047 (78.5)	286 (21.5)	0.211	0.646
	Yes	23 (82.1)	5 (17.9)		
Body mass	< 18.5	7 (0.7)	7 (1.7)	1.228	0.128
	18.5-24.9	383 (35.9)	115 (39.2)		
	25 – 29.9	481 (45)	122 (41.6)		
	>30	197 (18.4)	51 (17.4)		
Blood group	A	390 (77.5)	113 (22.5)	1.37	0.711
	B	255 (78.2)	71 (21.8)		
	O	328 (79.2)	86 (20.8)		
	AB	97 (82.2)	21 (17.8)		
History of baking bread	No	1006 (79.4)	261 (20.6)	6.66	*0.010
	Yes	64 (68.1)	30 (31.9)		

* is significant

The result revealed that 78.6% of patients had short MV, and 21.4% had LTMV. About 1.4% of patients underwent cardiopulmonary resuscitation in the first 24 h after surgery, which led to 66.7% of deaths. 99.9% of patients used nitroglycerin, 0.4% nitroprusside, and 0.2 labetalol to reduce blood pressure (Table 2).

**Table 2:** Frequency and percentage of patients' clinical status after surgery

Variables		Frequency	Percent
Status of mechanical ventilation	Short-term mechanical ventilation	1070	78.6
	Long-term mechanical ventilation	291	21.4
Cardiopulmonary resuscitation in the first 24 h	No	1343	98.6
	Yes	18	1.4
The result of cardiac resuscitation	alive	6	33.3
	death	12	66.7
Blood pressure medications	Nitroglycerin	1352	99.3
	Nitroprusside	6	0.4
	Labetalol	3	0.2
Cardiac medications	No	1066	78.3
	Epinephrine	211	15.5
	Norepinephrine	12	0.9
	Dopamine	1	0.1
	Dobutamine	5	0.4
	Epinephrine-Dobutamine	5	0.4
	Epinephrine-Norepinephrine	49	3.6
	Epinephrine-Norepinephrine-Dobutamine	9	0.7
	Epinephrine-Norepinephrine-Dopamine	1	0.1
	Dobutamine-Milrinone	1	0.1
	Epinephrine-Norepinephrine-Milrinone	1	0.1

Based on the results of the multivariate logistic regression test, the most important variables affecting the duration of MV were as follows: patients who had a history of baking bread had 2.7 times longer duration of MV (*P* = 0.004); patients with a history of COPD had 6.2 times longer duration of MV (*P* = 0.025); patients with a history of kidney disease had 1.2 times longer duration of MV (*P* = 0.036); patients with an intra-aortic balloon pump (IABP) had three times longer duration of MV (*P* = 0.030). Likewise, patients with higher creatinine levels after surgery had 6.8 times longer duration of MV (*P* = 0.004). The number of chest secretions in the first and second hours after surgery and cardiac enzymes (CK- MB) before surgery in patients also had a significant effect on the duration of MV. The number of breaths in 24 h after surgery, systolic blood pressure after surgery, diastolic blood pressure after surgery, creatinine level before surgery, central venous pressure after surgery, and ejection fraction before surgery were also protective factors (Table 3).

**Table 3:** Predicting LTMV based on the demographic and clinical variables by logistic regression

Variables		B	*P*-value	OR	CI
Age		0.010	0.381	1.01	0.988-1.03
Smoking history	No Yes	- 0.239	- 0.265	- -1.3	- 0.834-1.9
Usage history Narcotics	No Yes	- 0.190	- 0.439	- 1.2	- 0.743-1.9
History of baking bread	No Yes	- 1.005	- *0.004	- 2.7	- 1.4-5.4
History of heart disease	No Yes	- -0.056	- 0.779	- 0.945	- 0.637-1.4
History of pulmonary disease	No Yes	- 0.232	- 0.468	- 1.3	- 0.674-2.3
History of COPD	No Yes	- 1.8	- *0.025	- 6.2	- 1.2-31.06
History of kidney disease	No Yes	- 0.763	- *0.036	- 2.1	- 1.04-4.3
History of diabetes	No Yes	- 0.003	- 989.0	- 003.1	- 0.683-1.4
Anesthesia method in the operating room	Benzodiazepine Narcotics Etomidate	- 0.113	- 0.989	- 1.003	- 0.683-1.4
Use of intra-aortic balloon pump	No Yes	- 1.08	- *0.030	- 3	- 1.1 -7.8
Using a cardiopulmonary pump	No Yes	- 0.619	- 0.146	- 1.8	- 0.806-4.2
Valve surgery	No Yes	- 0.634	- 0.244	- 1.9	- 0.649 – 5.4
Respiratory rate 24 h after surgery		-0.921	*0.001	0.398	0.341 -0.465
Systolic blood pressure 24 h after surgery		-0.079	*0.001	0.924	0.885 -0.965
Diastolic blood pressure 24 h after surgery		-0.051	0.084	0.950	0.896 -1.007
Heart rate before surgery		0.018	0.074	1.01	0.998 -1.04
Heart rate 24 h after surgery		0.009	0.337	1.01	0.990 -1.02
Creatinine level before surgery		-1.5	*0.039	0.221	0.053-0.929
Creatinine levels 24 h after surgery		1.8	*0.004	6.4	1.8-22.8
Chest secretions in the first 12 h after surgery		0.001	*0.001	1.001	1-1.001
Chest secretions 12 h after surgery		0.001	*0.001	1.001	1-1.001
Central venous pressure 24 h after surgery		-0.271	*0.006	0.762	0.629 -0.924
Ejection Fraction rate before surgery		-0.035	*0.002	0.965	0.944-0.987
Cardiac enzymes (MB-CK) before surgery		0.124	*0.001	1.1	1.07 -1.2

## Discussion

In the present study, 21.4% of patients who underwent cardiovascular surgery were LTMV. One out of every five people who underwent heart surgery had MV longer than 24 h. In other studies, including the study of Trouillet et al. (2009), this rate was 6.2% [18]. In the study by Rajakaruna et al. (2005), the rate was 2.6% [19]. Also, Sharma and colleagues reported the rate of LTMV between 6 and 7% [20]. The possible reason for the discrepancy in the results can be due to the difference in the number of factors examined, the number of samples, the existence of interference between variables, the method of anesthesia, the method of operation, and the quality of care after surgery, which needs further investigation. In Iran and many medical centers, despite achieving the clinical weaning criteria, patients are subjected to MV for long hours without needing a ventilator from a respiratory therapist's viewpoint. Therefore, a standard national protocol for weaning from MV after cardiovascular surgery seems necessary.

The present study showed a significant relationship between age and the time of MV so this time was longer in older people, which is in line with the results of most studies conducted in this field. Also, in the current study, the average age was 61.9 years. In the study by Gomes et al. (2015), the average age of the people was 60.7 [6]. Serrano et al. (2005) reported the average age of the people to be 64 years [21]. Hasanzadeh et al.'s study (2017) also reported that the average age of people was 62.9 years [17]. Elderly people probably experience more medical conditions. Therefore, more attention is recommended in the care of this group. Since the nurses of the heart surgery units have an effective role in examining and recognizing patients' problems in the initial examination before accepting this group, it is advised to pay attention to the risk factors that increase the patient's problems with increasing age.

In the present study, people with a history of baking bread had a higher risk for LTMV (*P* = 0.010). The results of the searches showed that a similar study had not investigated the effect of baking bread on MV. Wood, animal dung, crop residues, and coal, typically used in fires or poorly functioning gas stoves, may lead to severe indoor air pollution. In Iran, most households in rural communities use biomass fuel for cooking, and the type of fuel used plays an important role in health and disease. This role becomes more important when cooking is done in the living room, which is common in winter [22].

In the current study, the variables of kidney disease, intra-aortic balloon pump, chronic obstructive pulmonary disease, creatinine level, chest secretions, ejection fraction, and cardiac enzyme level were predictors of long-term ventilation.

In the study of Alejandro et al. (2019), there was a significant relationship between the history of kidney disease and LTMV [23]. In the study of Totonchi et al., a significant association was observed between the history of kidney disease and the duration of MV after heart surgery [24]. The results of these studies are consistent with our study. In comparison, Imanipour et al. (2006) did not observe a significant relationship between the history of kidney disease and the time of weaning in patients undergoing bypass surgery [25]. In Judy et al.'s study (2001), there was no significant relationship between the history of kidney disease and weaning time [26]. The reason for these differences can be related to the number of cardiopulmonary pumps (CPB). Due to the harmful effects of the artificial CPB on liver function and renal filtration and the occurrence of changes in the pharmacokinetics of anesthetic drugs, such as pre-operative disease suffered from liver and kidney problems, the average duration of MV after the operation would increase. Hessel et al. (2019) mentioned a statistically significant relationship between LTMV and the use of an intra-aortic balloon pump. And people who had an intra-aortic pump had a higher chance of LTMV [27]. Also, Prapas et al. (2007) reported a significant relationship between LTMV and the use of an intra-aortic balloon pump [28].

Furthermore, Sharma et al. (2017) showed a significant relationship between the history of chronic obstructive pulmonary disease and prolonged MV [20]. The study by Faghani et al. (2017) also indicated that having a respiratory disease has a statistically significant relationship with the length of time the tracheal tube remains in cardiac patients [29]. Also, the study by Fitch et al. (2013) showed that there is a statistically significant relationship between the history of chronic obstructive pulmonary disease and the duration of separation from mechanical ventilation in patients after cardiovascular surgery, and a lack of these disorders has been reported as a protective factor [30]. There is a possibility that many people with respiratory disorders have reduced lung volumes. However, due to the lack of specific clinical symptoms, they are not subjected to more detailed lung examinations and are not reported as people with respiratory diseases. Therefore, it seems that patients with respiratory disorders, due to the decrease in lung capacities and inability to coordinate with MV after surgery, need more attention from the medical staff and the cardiac surgery intensive care unit nurses.

In line with the results of our study, in Totonchi et al.'s study, a significant relationship was observed between the amount of serum creatinine and kidney disease with the duration of weaning [24]. Also, Reddy et al. (2007) showed that pre-operative serum creatinine level is an independent risk factor for LTMV after heart surgery in adults [31]. Therefore, taking the necessary measures to reduce the amount of mechanical ventilation in patients can be effective against kidney damage and changes in creatinine levels. Siddiqui et al. (2012) showed that individuals with higher secretion rates were more likely to be on LTMV [32]. In the study of Jafarudi et al. (2014), the relationship between the number of chest secretions after the operation, as a quantitative variable, with the duration of MV indicated a meaningful positive correlation between these two variables [33]. Since the establishment of proper chest drainage with the appropriate discharge of secretions prevents pressure on the heart and lungs and improves the functioning of these organs, this improves the patient's breathing, reduces the need for ventilation support, and reduces the duration of MV. However, this issue, LTMV with increasing secretions, might be related to other factors, such as bleeding, which need further studies.

The results of different studies were in line with our study [18, 23, 34]. Regarding the ejection fraction (EF), in people with EF < 30%, compared to those greater than 30%, it was associated with a higher risk for longer MV. Disturbance in the function of the left ventricle leads to the aggravation of heart problems during surgery. In addition, the use of MV reduces the efficiency of the left ventricle and causes a decrease in end-diastolic and cardiac output volume, ultimately causing a reduction in cardiac preload and afterload. Therefore, patients with left ventricular dysfunction and low ejection fraction need more time to reach hemodynamic stability, so they achieve weaning criteria in a longer time, and they should be kept on MV for a longer time. No similar studies were found about the effect of cardiac enzymes on LTMV.

From the present study's findings, the number of breaths in 24 h after the operation has a statistically significant relationship with the duration of MV and acts as a protective factor. By increasing each unit of RR, LTMV decreases by 0.921 h. Jafroudi et al. (2014) also observed a significant relationship between the number of breaths and the duration of MV [33]. Also, the results of our study conform with the results of Annapoorna's research, which concluded that patients are at risk of increasing the duration of MV with a decrease in breathing rate [35]. Furthermore, in the study of Subic and colleagues, it was concluded that the ratio of respiratory rate to tidal volume is a sensitive indicator for predicting the outcome of weaning from MV [36]. It seems very high or low respiratory rate leads to a disturbance in the patient's hemodynamic and physiological condition, which can play an important role in the outcome of patients' weaning.

Another finding of the present study was that postoperative systolic blood pressure has a statistically significant relationship with the duration of MV and acts as a protective factor. The results of the study by Siddiqui et al. (2012) also showed that people with hypertension had a higher chance of LTMV [33]. Yeganeh et al. (2018) have also reported a significant positive relationship between blood pressure changes in patients until the time of endotracheal tube removal [37]. Considering that blood pressure is directly related to the amount of ventilation, it can be understood that if decreases or an increase in blood pressure, the volume of blood in the exchange with air in the lungs changes, and as a result, it will have a direct effect on the ventilation rate.

## Conclusion

The results of this study showed that the rate of LTMV was 21.4%. It is recommended to consider the affected factors such as the history of baking bread, history of pulmonary diseases, history of kidney disease, use of the intra-aortic balloon pump, respiration rate 24 h after surgery, systolic blood pressure 24 h after surgery, creatinine level before surgery, creatinine level 24 h after surgery, chest secretions after surgery, ejection fraction before surgery and cardiac enzymes (CK- MB). Considering the factors that affect the prolongation of MV, it is possible to decrease its duration in patients through more control and monitoring, regular implementation of standard weaning protocols, and accomplishing training programs for personnel working in the intensive care unit. This measure reduces and prevents physical and mental side effects, the extended stay of these patients in the intensive care unit, and costs.

## Data Availability

Data available by contacting the corresponding author.

## Abbreviations

MV: Mechanical ventilation
SD: Standard deviation
LTMV: Long-term mechanical ventilation
